# Language and Memory Improvements following tDCS of Left Lateral Prefrontal Cortex

**DOI:** 10.1371/journal.pone.0141417

**Published:** 2015-11-03

**Authors:** Erika K. Hussey, Nathan Ward, Kiel Christianson, Arthur F. Kramer

**Affiliations:** 1 Department of Psychology, University of Illinois at Urbana-Champaign, Champaign, Illinois, United States of America; 2 Department of Education Psychology, University of Illinois at Urbana-Champaign, Champaign, Illinois, United States of America; 3 Beckman Institute for Advanced Science and Technology, University of Illinois at Urbana-Champaign, Urbana, Illinois, United States of America; University Medical Center Goettingen, GERMANY

## Abstract

Recent research demonstrates that performance on executive-control measures can be enhanced through brain stimulation of lateral prefrontal regions. Separate psycholinguistic work emphasizes the importance of left lateral prefrontal cortex executive-control resources during sentence processing, especially when readers must override early, incorrect interpretations when faced with temporary ambiguity. Using transcranial direct current stimulation, we tested whether stimulation of left lateral prefrontal cortex had discriminate effects on language and memory conditions that rely on executive-control (versus cases with minimal executive-control demands, even in the face of task difficulty). Participants were randomly assigned to receive Anodal, Cathodal, or Sham stimulation of left lateral prefrontal cortex while they (1) processed ambiguous and unambiguous sentences in a word-by-word self-paced reading task and (2) performed an *n*-back memory task that, on some trials, contained interference lure items reputed to require executive-control. Across both tasks, we parametrically manipulated executive-control demands and task difficulty. Our results revealed that the Anodal group outperformed the remaining groups on (1) the sentence processing conditions requiring executive-control, and (2) only the most complex *n-*back conditions, regardless of executive-control demands. Together, these findings add to the mounting evidence for the selective causal role of left lateral prefrontal cortex for executive-control tasks in the language domain. Moreover, we provide the first evidence suggesting that brain stimulation is a promising method to mitigate processing demands encountered during online sentence processing.

## Introduction

Almost all daily mental activities require some form of computational filtering to make sense of noisy input. Executive-control supports these efforts for higher-level cognitive tasks when we must resolve among multiple interfering sources of information. Specifically, it services the selection of goal-compatible information, allowing us to ignore irrelevant information, including default responses and input that we are biased to automatically process [1–3]. Consider, for example, the task of interpreting speech during a conversation. Listeners must quickly derive meaning from utterances by relying largely on stored linguistic and mnemonic knowledge. Searching this vast information space and integrating it with real-time input (a process referred to as incrementality; see [4]) often gives rise to situations when several eligible meanings exist. A comprehender’s ability to select among conflicting options is crucial for successful communication and relies largely on non-linguistic abilities, like executive-control [5–11]. Here, we focus primarily on executive-control for language use and ask whether using brain stimulation to target a cortical region reputed to support executive-control has concomitant effects on interpretation processes, namely when readers must choose among multiple possible meanings of a sentence.

Converging evidence from neuropsychological patients and neuroimaging in healthy adults indicates a supportive role of left lateral prefrontal cortex (LPFC) in executive-control conditions on tasks in the memory and language domains [12–16]. Patients with focal lesions to left LPFC demonstrate deficits limited to language processing and recognition memory scenarios requiring irrelevant information to be ignored [9,17–19]. In sentence processing, for example, left LPFC patients struggle to correctly arrive at the intended meaning of ambiguous sentences due to their inability to engage executive-control to ignore one (incorrect) interpretation in favor of another (correct) one [9]. Remarkably, sentences without temporary conflict or ambiguity are processed and comprehended with little issue, even when they contain complex syntax that requires additional processing [20], but see [21–22]. The selective nature of these findings extend to the memory domain, such that left LPFC patients consistently show exaggerated effects for familiar-but-irrelevant recognition probes in a recognition memory task, while probes with minimal interference do not suffer [23–24]. Additionally, two new functional neuroimaging findings in healthy adults provide compelling correlational evidence linking left LPFC to executive-control conditions in the memory and language domains. In one instance [25], neural activation during the Stroop task (when a color word is in conflict with its font color) correlated with activation levels *in the same participants* associated with processing ambiguous sentences like “Clean the frog with the leaf,” where “leaf” can be an instrument (use the leaf to clean the frog) or a modifier (clean the frog that has a leaf). In another study [26], co-localization of activity in left LPFC appeared for the Stroop task, the Flanker task (target arrow direction is in conflict with distractor arrows), and reading Chinese sentences that elicited temporary ambiguity among meanings. Alongside the patient results, co-recruitment patterns indicate that left LPFC subserves a variety of memory and language tasks, suggesting a shared, process-specific role of executive-control across both domains [8–9].

One implication that follows from these findings involves improving language use through interventions that target executive-control. Indeed, preliminary evidence indicates that practice on executive-control tasks leads to improvements on untrained measures of syntactic ambiguity resolution [27–28]. Following multiple weeks of exposure to a performance-adaptive *n*-back task containing interference lures, trainees were faster to read and more accurate to comprehend syntactically ambiguous sentences. Importantly, other untrained task conditions with minimal executive-control demands did not result in the same improvements after training, and other training groups (that trained on tasks unrelated to executive-control) demonstrated no improvements. Given that the training task (*n*-back-with-lures) and the improved transfer measure (syntactic ambiguity resolution) recruit left LPFC, it is possible that training honed left LPFC resources, which cascaded into benefits for untrained tasks relying on the same cognitive mechanisms (for similar shared-resource/process-specific arguments, see [27–31]).

To further probe this possibility, we use a non-invasive brain stimulation approach to change cortical excitability in left LPFC and assess the behavioral consequences on syntactic ambiguity resolution and performance on the *n*-back task. If *n*-back-with-lures and syntactic ambiguity resolution tap the same cognitive mechanism, and if that mechanism is supported in part by left LPFC regions (as the evidence reviewed above suggests), then we would expect tDCS over left LPFC to influence only conditions with elevated executive-control demands in each task. That is, in the present study, we entertain the possibility that tDCS may be a method for testing linking hypotheses between training and transfer measures for future intervention studies that are guided by process-specific principles [29]. In the next section, we describe our brain stimulation method and highlight relevant tDCS findings that delineate the functional role of left LPFC for a range of executive-control measures. We argue that tDCS is a promising tool to assess the causal role of left LPFC-mediated executive-control for language.

## Brain Stimulation of Executive-Control Regions

There has been a recent influx of studies aimed at temporarily affecting cognition with non-invasive brain stimulation. Here, we focus on transcranial direct current stimulation, or tDCS, a technique that involves sending a small electrical current (1–2 milliamperes, mA) through the scalp from an anode to a cathode with the goal of altering neuronal excitability. Specifically, cortical regions near the anode have shown increased activity due to temporary neuronal depolarization, while regions close to the cathode have shown reduced activity due to acute neuronal hyperpolarization [32–34]. Typically stimulation is accomplished by passing current through two saline-soaked sponges that vary in size from 11cm^2^ to 35cm^2^ in size; the current study implemented a “high definition” approach by administering the current with two 1.3cm^2^ EEG-like electrodes, which has been shown to increase precision and decrease current shunting [35–36].

While not completely ubiquitous [37–39], the facilitatory effects of anodal tDCS have been demonstrated across a variety of cognitive processes [40], including visual perception [41], attention [42], executive function [43–45], learning [46–47], problem solving and planning [48–49], and recognition [50–53]. Relevant for the current study, anodal tDCS has produced several instances of improvement on tasks tapping executive-control. For example, participants show smaller interference effects on the Stroop task following anodal stimulation over left or right LPFC for 20 minutes compared to sham controls [54–55]. Similarly, compared to sham stimulation, 10 minutes of anodal stimulation over left LPFC resulted in faster responses on a recent-negatives Sternberg recognition memory task [56]. Finally, Ohn and colleagues compared sham stimulation to anodal tDCS over left LPFC and found higher accuracy on a 3-back task [57]. Pertinent to the latter effect, a recent review of tDCS and *n*-back studies reported improved performance across 33 experiments for anodal tDCS over prefrontal regions compared to sham stimulation [58]. To evaluate the role of left LPFC for executive-control demands in *n-*back, we included a modified version of the *n*-back task containing interference lures [59–60].

In addition to the executive-control benefits of anodal stimulation over LPFC in the memory domain, there are also many reports of tDCS-mediated improvements in language processing, including artificial grammar learning [61], verbal categorization [62], reading efficiency [63], and language production among healthy adults and individuals with aphasia [64]. Many of these findings involve stimulating temporal regions, but those involving stimulation of left LPFC generally result in quicker production times to picture cues [65–68], better semantic fluency [69–71], faster proper naming [72], and fewer speech errors [73–74]. Such production-based facilitation effects are accompanied by decreased left LPFC activation [68], increased functional connectivity within the default mode network [72], and reduced activity in the delta frequency band [75] (see [76] for complementary evidence in the alpha band for the *n*-back task). These findings suggest that the improved mechanism may be inhibitory in nature (i.e., a component of executive-control) [74].

Alongside the converging evidence for the role of left LPFC for executive-control, and in light of the extant evidence for tDCS-mediated improvement in executive-control and language processing, we anticipate anodal stimulation of left LPFC to positively influence sentence comprehension and real-time interpretation efforts when readers must resolve syntactic ambiguity. Promising relevant psycholinguistic data exist for sentence production [73] and idiom comprehension [77] such that both tasks benefit from acute left LPFC stimulation. To our knowledge, however, the present study constitutes the first effort to test for the effects of left prefrontal tDCS in the *sentence comprehension* domain.

## Materials and Methods

### Participants

Seventy-nine participants were assigned to receive anodal, cathodal, or sham tDCS over left LPFC. Twenty-seven participants received anodal stimulation (12 females; M: 19.5 years; range: 18–23 years), 26 received cathodal stimulation (11 females; M: 20.2 years; range: 18–28 years), and 26 received sham (12 females; M: 20 years; range: 18–25 years). Stimulation was administered in a single-blind design, such that participants were unaware of their group assignment. All participants reported being right-hand dominant, fluent English speakers, and no one had any prior experience with tDCS.

### Design

**Fig 1:**
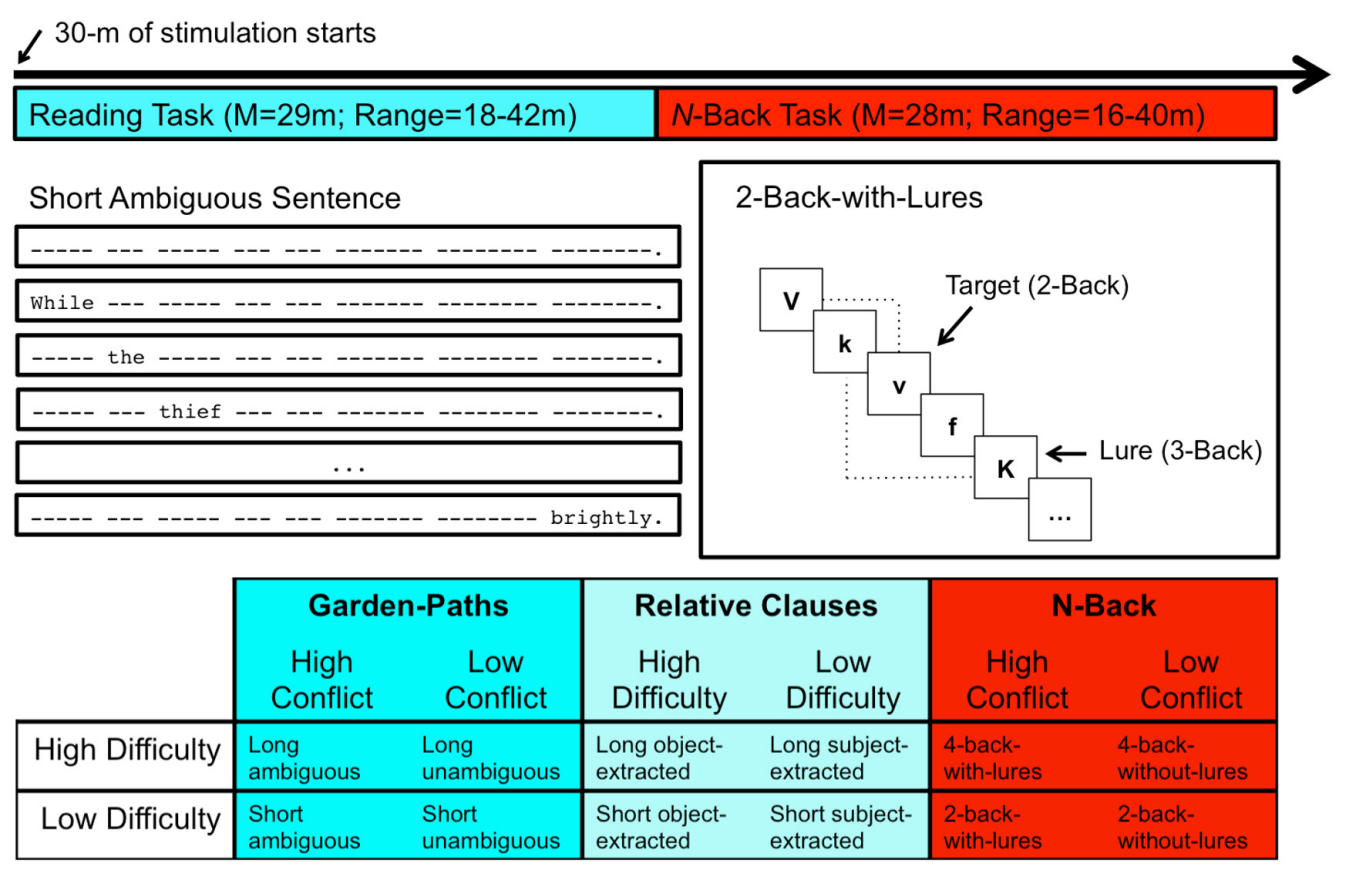
Experimental design. Timeline of task administration and design details of each task.

Following tDCS electrode application, all participants completed two behavioral tasks in the language (Reading Task) and memory (*N*-Back Task) domains (see Fig 1). All participants completed the reading task first (time-on-task M = 29m, SD = 6.3m, range: 18–42m) followed by the *n*-back task (time-on-task M = 28m, SD = 3.7m, range: 16–40m). The stimulation period began at the onset of the reading task and lasted for 30 minutes. Because the tasks were self-paced, subjects received different amounts of simultaneous (or “online”) stimulation while performing the tasks. More than half of the subjects (n = 51) experienced stimulation for the entirety of the reading task, with 38 of these participants also receiving stimulation during the *n*-back task. Importantly, given that the effects of tDCS can persist for up to 90 minutes beyond active stimulation episodes [32,78], the stimulation was expected to modulate performance on both tasks. All participants finished the study by filling out a questionnaire that assessed expectations of stimulation group assignment and other experiences throughout the session.

### tDCS Protocol

Two high-definition electrodes were placed on the left side of the head to send a current through only the left hemisphere, targeting left LPFC. The polarity of the current minimally differed in the Anodal and Cathodal groups, such that the anode was placed over left LPFC (cathode over left occipital cortex) for participants in the Anodal group and the anode over left occipital cortex (cathode over left LPFC) for those in the Cathodal group. Participants assigned to receive sham stimulation always had an anode over left LPFC and cathode over left occipital cortex.

#### Apparatus

Stimulation was delivered via a Soterix Medical 1x1 Transcranial Direct Current Low-Intensity Stimulator (Model 1300A). Anode and cathode cables were connected to the device and 1.3cm^2^ high-definition Ag/AgCl electrodes [79] were attached. Modular electroencephalogram caps manufactured by EasyCap were outfitted with electrode holders over sites F3 and O1 according to international 10–20 standards [80] into which the electrodes were placed for stimulation.

**Fig 2:**
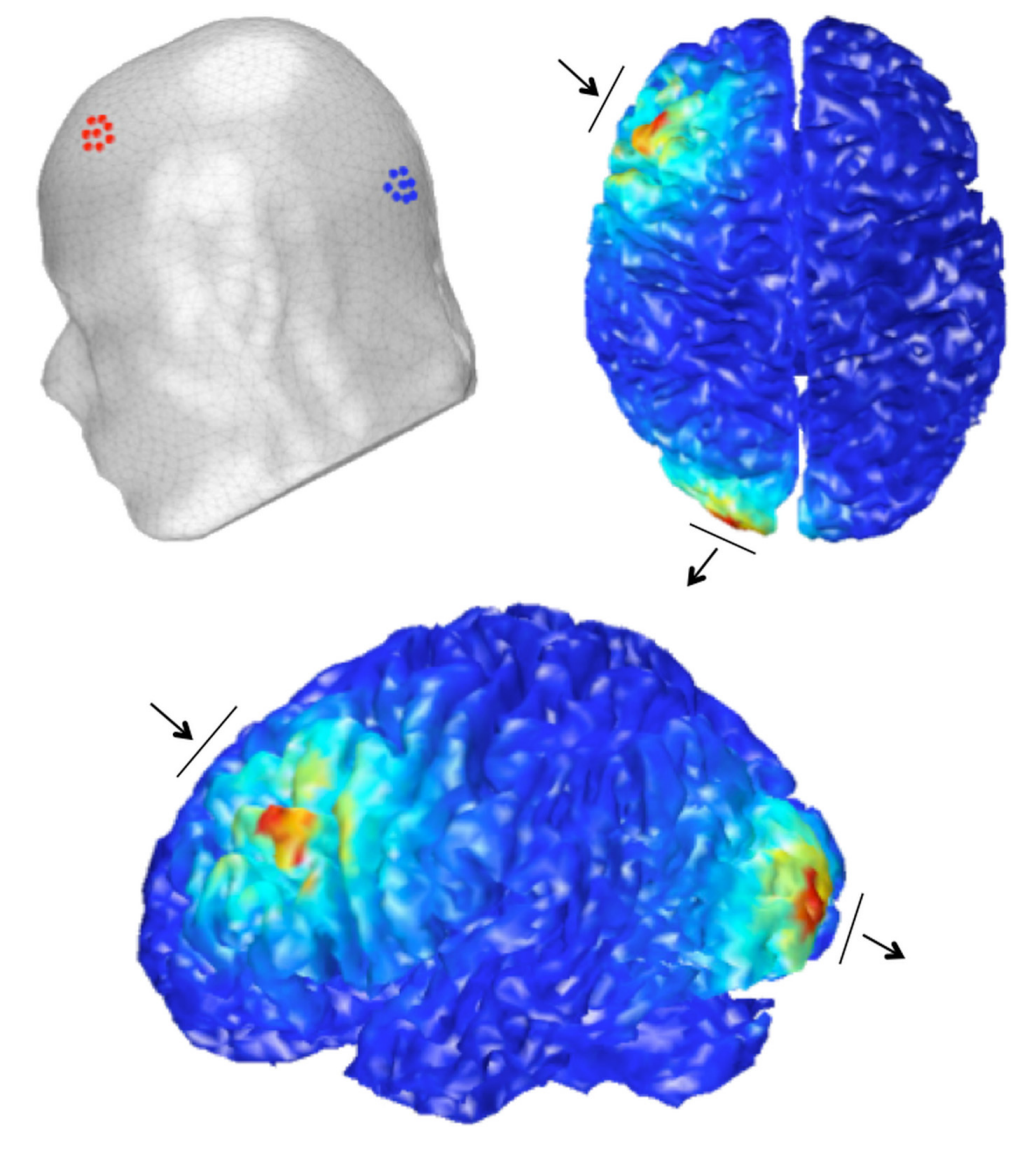
Modeled stimulation electric field. Electrode placement locations (anode in red and cathode in blue) and the output of finite element modeling of the estimated electric field for the Anodal stimulation condition. Arrows denote the direction of the current from anode to cathode.

We analyzed the local electric field generated through the brain with 3D finite element modeling as a function of our selected electrode sites using the COMETS toolbox for MATLAB [81]. Fig 2 shows the estimated cortical stimulation current for the anodal and cathodal groups based on a standard Montreal Neurological Institute (MNI) human head model. The change in cortical excitability between the two montages demonstrates that there is minimal bridging between electrodes such that stimulation is primarily restricted to left lateral prefrontal and left occipital regions.

#### Procedure

The University of Illinois, Urbana-Champaign Institutional Review Board approved all procedures used. All participants signed an informed consent document and were told that the study involved “high-definition” electrode montages to precisely target brain regions by sending a weak current through two small electrodes on the left side of the head. All participants’ head circumferences were measured from the inion to the nasion to select the appropriate cap size. The electrode cap was positioned such that front and back midline edges of the cap were 1 centimeter from the inion and 3 centimeters from the nasion, respectively. Electrode sites were prepared with highly conductive gel (SignaGel, Parker Laboratories, Fairfield, NJ), and electrodes were attached to the tDCS device.

Participants in the Anodal and Cathodal stimulation groups received 2.0 mA current for a full 30-minute period. Stimulation was ramped up and ramped down over 30 seconds at the beginning and end of each session, with continuous current applied for 30 minutes. Participants in the Sham group underwent all of the same procedures as those in the Anodal and Cathodal groups except that the current lasted only 30 seconds at the start and end of the session. The comparable ramp-up/ramp-down sequence for Sham controls elicits the same sensation to equalize expectations about whether stimulation was applied, but does not induce any substantial neuronal effects [82–83]. All participants experienced a short exposure period to acclimate to the stimulation sensation before the longer stimulation period began, and no participants reported any discomfort or pain throughout the session.

### Reading Task

To assess the selective role of left LPFC-mediated executive-control for sentence processing, participants read syntactically ambiguous and unambiguous sentences (Sentences 1 and 2 in Table 1), embedded among object- and subject-extracted relative clause sentences (Sentences 3 and 4 in Table 1; see S1 Table for all stimulus sentences). Ambiguous sentences like 1a and 1b invoke executive-control due to their temporary ambiguity [8]. Specifically, the verb “hid” can be used transitively (the thief is hiding the jewelry) or reflexively (the thief is hiding himself). Although the correct analysis of this sentence is unknown until late arriving information appears that signals the reflexive interpretation (“sparkled brightly”), native English readers strongly prefer a transitive interpretation initially, because “jewelry” is a semantically viable object that thieves might hide [84–86]. That is, on-the-fly sentence processing lures readers down the “garden-path” to expect one interpretation (the transitive), when in fact, another is ultimately revealed to be correct (the reflexive). Executive-control supports such syntactic ambiguity resolution by allowing readers to inhibit their initial preferred transitive interpretation in favor of the correct reflexive meaning [27,87–89]. Sentences like 2a and 2b in Table 1 reverse the clause order, removing any temporary ambiguity, and thus, the need to use executive-control to resolve among interpretations is diminished.

**Table 1:** Example stimulus sentences for each condition.

	Sentence	Conflict Level	Difficulty Level
1a	While the thief /hid /the jewelry that was elegant and expensive /**sparkled brightly**.	High (Ambiguous)	High (Long)
1b	While the thief /hid/ the jewelry /**sparkled brightly**.	High (Ambiguous)	Low (Short)
2a	The jewelry that was elegant and expensive/ **sparkled brightly** /while the thief /hid.	Low (Unambiguous)	High (Long)
2b	The jewelry /**sparkled brightly** /while the thief /hid.	Low (Unambiguous)	Low (Short)
3a	The farmer /**who the expert questioned that was outgoing and enthusiastic** /promoted /the product /at the fair.	Low	High (Long OE)
3b	The farmer /**who the expert questioned** /promoted /the product /at the fair.	Low	High (Short OE)
4a	The farmer /**who questioned the expert that was outgoing and enthusiastic** /promoted /the product /at the fair.	Low	High (Long SE)
4b	The farmer /**who questioned the expert** /promoted /the product /at the fair.	Low	Low (Short SE)

Example Garden-Path (1–2) and Relative Clause (3–4) sentences. Slashes indicate sentence region boundaries used for reading time analyses, where bold denote the regions of interest. OE = object-extracted; SE = subject-extracted

Interestingly, elongating the ambiguous portion of the sentence (prior to the disambiguating information, “sparkled brightly”) gives rise to larger garden-path effects, due to a mounting incremental processing commitment [84] (Sentence 1b in Table 1). Within the context of a word-by-word self-paced moving-window reading paradigm (see description below), elongating the ambiguous sentence region also places a premium on other cognitive demands, like working memory [90]. As a result, this manipulation introduces task-level complexity that may be separable from the executive-control demands encountered in ambiguous sentences. Thus, if active stimulation of left LPFC influences executive-control alone, the Anodal group should outperform the Cathodal and Sham groups only on ambiguous items, regardless of sentence length prior to the introduction of disambiguating information (at “sparkled brightly”).

To further test the selectivity of executive-control in sentence processing, we also included relative clause sentences, which incur processing costs due to cognitive demands largely separable from executive-control [91]. Specifically, reading times in the embedded relative clause region of object-extracted items (the bolded section in Sentences 3a and 3b in Table 1, “who the expert questioned”) are typically longer relative to the comparable region in subject-extracted sentences (the bolded section in Sentences 4a and 4b in Table 1, “who questioned the expert”). This increased processing difficulty has been explained in terms of greater syntactic complexity due to integration costs, such that the representation associated with the subject noun phrase (“the farmer”) may partially decay before a reader encounters the verb (“promoted”; [92–93]). Furthermore, and relevant to the current claim, there is no association between left LPFC and comprehension of syntactically complex sentences like relative clauses among patients with circumscribed damage to this region [94], yet injury to left LPFC reliably predicts failure to recover from garden-path misinterpretations [9,19,87]. Therefore, Anodal stimulation of left LPFC should *not* influence processing under all states of effortful sentence processing, but rather only when executive-control demands are high (i.e., when one seemingly correct interpretation must be inhibited in favor of another).

#### Materials

Participants read a total of 144 sentences. Twenty-four verbs were used that contained the transitive/reflexive ambiguity and 2 distinct contexts were created for each verb to create a total of 48 unique sentence frames. Four versions of each sentence were created to vary minimally in terms of Conflict Level (ambiguity) and Difficulty Level (length), resulting in 12 sentences for each of the 4 conditions (e.g., Sentences 1a, 1b, 2a, 2b in Table 1). We created 4 lists by Latin-squaring conditions, so as to not include a repeating frame within a list. For example, if List 1 contained a short ambiguous version of a sentence, then List 2 would contain the long ambiguous form, List 3 the short unambiguous sentence, and List 4 the long unambiguous version. Similarly, 48 unique relative clause sentences were used that varied minimally in terms of subject- or object-extraction and length (short or long), resulting in 12 sentences for each of the 4 conditions (e.g., Sentences 3a, 3b, 4a, 4b in Table 1). The same Latin-square approach was applied to these materials, as well, to create 4 sets within each of the 4 lists. We fully counterbalanced list administration across participants to ensure equal numbers of observations for each item. The 96 critical constructions were embedded within 48 additional filler sentences, which did not involve garden-path recovery or relative clause extractions, and contained a range of structures to conceal both manipulations. All items were pseudorandomized within each list to prevent any within-condition repeats on back-to-back trials. An exhaustive list of the critical items (garden-path and relative clause sentences) can be found in the S1 Table.

#### Procedure

Participants read sentences via a non-cumulative, self-paced moving-window design; each word was shown one at a time as the subjects pressed a button until all words in a sentence were presented just once [95]. Sentences appeared on a single line with a dash replacing all letters and punctuation, with spaces between words preserved (see Fig 1). As participants pressed buttons, the dashes corresponding to the current word disappeared to reveal that word. Upon pressing the "Next" button, the word was masked with dashes and the following word appeared. Participants were not allowed to revisit words of the sentence after they appeared. Following the final word in the sentence, a yes/no comprehension question appeared on a new screen. For the garden-paths, these questions probed for the reflexive interpretation (e.g., “Did the thief hide himself?” for Sentences 1 and 2 in Table 1); therefore, an incorrect ‘no’ response indexed offline misanalysis and a correct ‘yes’ response indexed successful revision [84,96]. As a result, for all ambiguous and unambiguous sentences, the correct answer was always ‘yes.’ For the relative clauses, the questions gauged comprehension of information unrelated to the embedded clause interpretation (e.g., “Was the product promoted on TV?” for Sentences 3 and 4 in Table 1). Across all items in a list, yes/no responses were equally likely. We recorded accuracy to comprehension questions and word-by-word response times on each trial for later analysis.

### *N*-Back Task

Following the reading task, participants performed an *n*-back letter memory task during which they indicated when a current item matched an item presented *n* trials prior. Similar to the design of the reading task, our version of *n*-back minimally and parametrically varied Conflict Level and Difficulty Level. Difficulty was manipulated by changing the number of to-be-remembered items (*n-*level); for example, remembering 2 items back is easier than remembering 4 items back due to changes in processing demands that are distinct from executive-control [97]. Conflict was manipulated by introducing highly-familiar non-target stimuli (interference lures), which are known to engage left LPFC executive-control brain regions [98–100].

#### Procedure

Following a 500ms fixation cross, letters were displayed serially for 500ms with an ISI of 2s. All letters were drawn from a subset of phonologically distinct consonants (b, c, d, f, h, j, k, l, m, p, q, r, s, t, v, x) and were displayed in mixed upper- and lower-case to prevent participants form responding simply based on iconic memory. Participants indicated by button press whether the current letter, regardless of case, had appeared *n* items previously by pressing one of two keys corresponding to ‘Target’ or ‘Non-Target.’ Participants performed two blocks of 2-back followed by 2 blocks of 4-back. All sequences contained 20+*n* items, partitioned into 6 targets and 14+*n* fillers. The second half of the blocks at each *n*-level contained 6 interference lures and 8+*n* fillers. Lures were defined as items that repeated in positions *n*+1, *n*+2, *n*-1, and *n*-2 [28,60]. For example, during a 2-back task, the second appearance of *J* in the sequence *j*, *p*, *k*, *j* is considered a lure because it matches the identity of an item presented 3 trials prior instead of 2 trials prior (see Fig 1). All participants began the task with a practice 2-back sequence, followed by blocks of *n*-back in ascending difficulty: 2-back-without-lures, 2-back-with-lures, 4-back-without-lures, and 4-back-with-lures. Feedback in terms of accuracy and average response time was provided after each sequence. All participants performed five sequences of each block, and they were explicitly notified when the task transitioned from 2-back to 4-back, but not notified when lures were introduced at each *n*-level. We recorded accuracy and response time for each item in a sequence, as well as accuracy on entire sequences for later analysis.

## Results

### Statistical Analysis

Linear mixed-effects models (using R’s lme4.0 package version 1.17) were used to evaluate reading and *n*-back performance. We included Stimulation (anodal, cathodal, sham), Conflict Level (high, low), and Difficulty Level (high, low) as fixed (independent) factors in all of our models. We also included random effects of Subjects and nested random slopes for the fixed factors to account for participant variability to stimulation, conflict, and difficulty. For the reading measures, we also included a random intercept term for Items and nested random slopes of the fixed factors to account for item-level variability in conflict and difficulty [101]. For each dependent measure, we selected the maximal model that converged (i.e., the model containing the most allowable slope terms [102]). In all of the model summary tables, we report model coefficients, standard errors, and t- or z-values for each main effect and higher-level interaction. *P*-values were determined using a Kenward-Roger approximation [103]. When discussing each model below, we first describe task-level effects (i.e., main effects of Difficulty and Conflict and the interaction between Difficulty and Conflict), followed by any Stimulation effects. Each subject’s data are provided in S1 File.

#### Contrast coding

Within-subjects fixed factors (Conflict Level and Difficulty Level) were assessed via mean-centered orthogonal Helmert coding, which allowed us to examine the difference between factor levels while accounting for differences in the number of observations contributing to each factor level [104]. Contrast coding was determined on the basis of hypothesized effects: Positive values were assigned to factor levels expected to elicit worse performance. For example, because we expected 4-back to lead to worse performance than 2-back, Difficulty Level in the *N*-Back Task would be coded with a positive coefficient for 4-back (0.5) and a negative for 2-back (-0.5). Thus, a positive model coefficient for the main effect of *N*-Back Difficulty Level would signal worse performance on 4-back relative to 2-back, while a negative coefficient would suggest worse 2-back performance relative to 4-back. With respect to the remaining factors, we positively coded the following levels: lures of *N*-Back Conflict Level, ambiguous of Garden-Path Conflict Level, long of Garden-Path Difficulty Level, object-extracted of Relative Clause Sentence Type, and long of Relative Clause Difficulty Level. For more information on comprehensively interpreting effects on the basis of model coefficients, see [105].

To test for the effects of Stimulation, we implemented two models with dummy contrast coding that varied only in their baseline reference level. In this case, the reference level was compared to the remaining levels. For instance, the first model included the Anodal group as the reference level, which allowed us to compare Anodal vs. Sham and Anodal vs. Cathodal. Since Cathodal and Sham stimulation are not compared to one another in this contrast, we implemented a second contrast coding with the Sham group as the reference level, which gave us coefficients comparing Sham vs. Anodal and Sham vs. Cathodal. Combining two contrast codes allowed us to make comparisons of each group with every other group [106]. Since both contrasts share one redundant comparison (Anodal vs. Sham), we report only the model coefficients for this comparison from the first contrast (with Anodal as the reference level).

We expect effects for all three comparisons if Anodal stimulation of left LPFC improves executive-control and Cathodal stimulation compromises performance, such that Anodal stimulation should result in superior performance relative to the Sham and Cathodal stimulation, and the Sham group should outperform the Cathodal group. Moreover, if stimulating left LPFC has selective effects on executive-control, then we would only expect Stimulation to interact with Conflict Level (and not Difficulty Level). If Stimulation interacts with only Difficulty Level, this would indicate that left LPFC services cases of heightened difficulty, regardless of the need for executive-control; whereas, if Stimulation interacts with *both* Conflict Level and Difficulty Level, we would conclude that left LPFC supports both processing demands.

### Reading Performance

Reinterpretation abilities were assessed via comprehension accuracy and reading time. For both measures in garden-path sentences, we assessed Conflict Level (high: ambiguous sentences; low: unambiguous sentences) and Difficulty Level (high: long sentences; low: short sentences). For relative clauses, we assessed Difficulty Level with sentence type (high: object-extracted sentences; low: subject-extracted) and sentence length (high: long sentences; low: short sentences).

#### Garden-path comprehension accuracy

**Fig 3:**
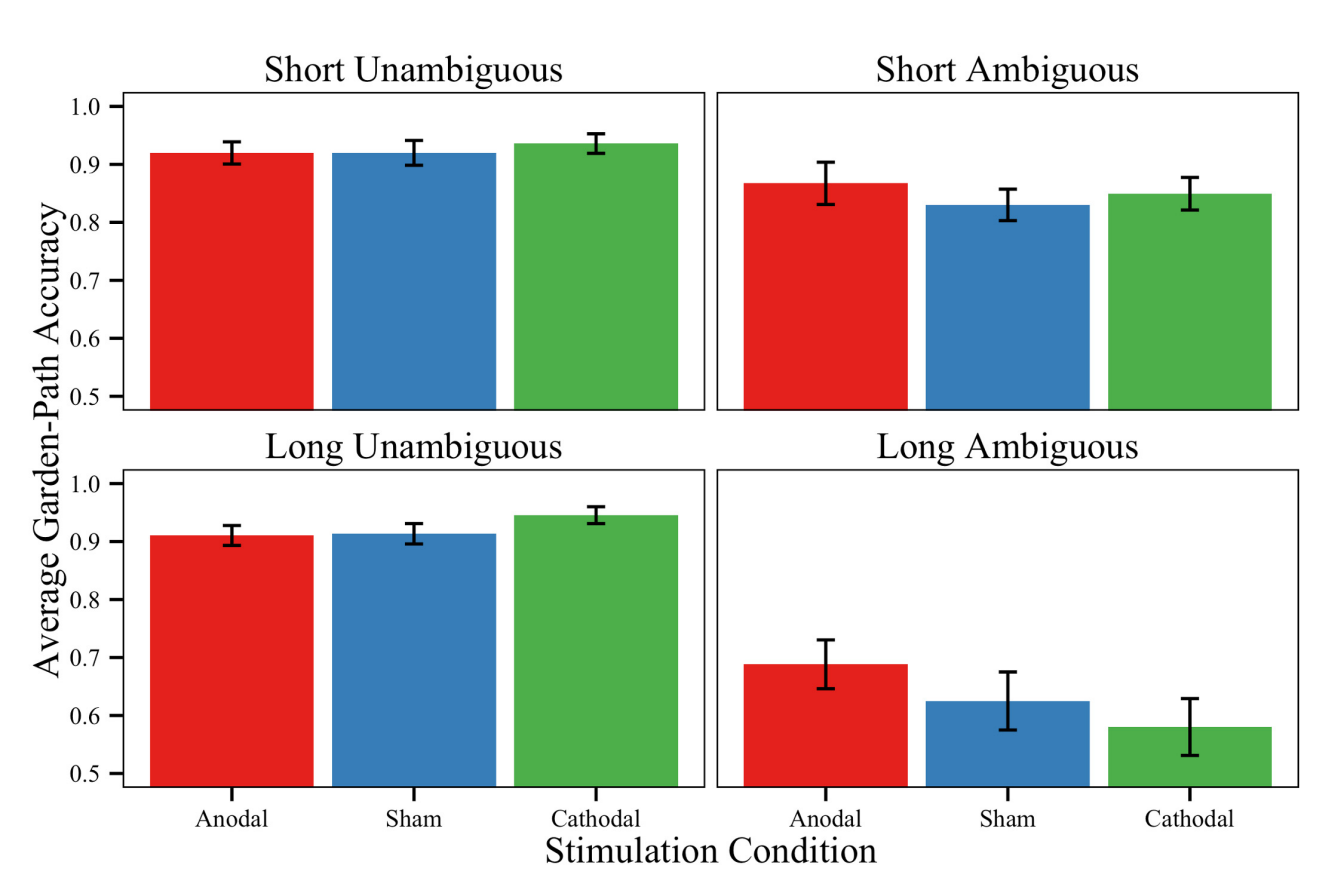
Garden-path comprehension accuracy. Average comprehension accuracy to questions following garden-path sentences for each stimulation group and task condition. Error bars = ±1 standard error of the mean.

Comprehension accuracy was modeled as a binomial variable using a generalized linear mixed-effects model (Table 2). Replicating prior garden-path findings [84, 85], we observed a main effect of Conflict Level and an interaction of Conflict Level and Difficulty Level (see Fig 3). Specifically, ambiguous sentences resulted in worse accuracy compared to unambiguous items (M_AM_ = 0.741 vs. M_UN_ = 0.924), an effect that was exaggerated in longer sentences (M_AM_ = 0.632 vs. M_UN_ = 0.923) compared to shorter ones (M_AM_ = 0.849 vs. M_UN_ = 0.925). We also observed a main effect of Difficulty Level, indicating that longer sentences resulted in worse average accuracy than shorter sentences (M_Long_ = 0.777 vs. M_Short_ = 0.887).

**Table 2:** Estimate coefficients from generalized linear mixed-effects models for garden-path accuracy on the reading task.

Predictor	Coefficient	SE	z-value
Fixed Effects			
**Intercept**	**2.589**	**0.267**	**9.686** *
**Conflict Level**	**1.526**	**0.316**	**4.826** *
**Difficulty Level**	**0.841**	**0.247**	**3.409** *
**Conflict Level x Difficulty Level**	**-1.227**	**0.456**	**-2.694** *
Anodal vs. Cathodal	-0.038	0.312	-0.123
Anodal vs. Sham	-0.219	0.308	-0.711
Sham vs. Cathodal	0.203	0.317	0.639
**Conflict Level x Anodal/Cathodal**	**0.925**	**0.378**	**2.449** *
Conflict Level x Anodal/Sham	0.459	0.367	1.251
Conflict Level x Sham/Cathodal	0.309	0.363	0.850
Difficulty Level x Anodal/Cathodal	0.045	0.335	0.133
Difficulty Level x Anodal/Sham	-0.002	0.323	-0.006
Difficulty Level x Sham/Cathodal	0.049	0.338	0.146
Conflict Level x Difficulty Level x Anodal/Cathodal	-0.521	0.609	-0.856
Conflict Level x Difficulty Level x Anodal/Sham	0.121	0.579	0.209
Conflict Level x Difficulty Level x Sham/Cathodal	-0.568	0.608	-0.934
Random Effects			
Subjects (Intercept)	0.9374		
Subjects (Conflict Level)	0.6324		
Subjects (Difficulty Level)	0.2587		
Subjects (Conflict Level x Difficulty Level)	0.0045		
Items (Intercept)	0.9154		
Items (Conflict Level)	0.5032		

Difficulty Level refers to the length of the ambiguous region (long vs. short) and Conflict Level refers to sentence ambiguity (ambiguous vs. unambiguous). Bold indicates coefficients that are significant. SE = standard error

*significant at the p<0.05 level

Interestingly, in terms of stimulation effects, the models revealed a significant interaction between Conflict Level and Stimulation for just the anodal/cathodal contrast. As predicted, the Anodal group demonstrated overall better accuracy on ambiguous items compared to the Cathodal group (M_Anodal_ = 0.778 vs. M_Cathodal_ = 0.715). No effects emerged for the remaining contrasts, though the effect was numerically in the direction that polarity effects would predict; the Anodal group was more accurate on ambiguous items than the Sham group (M_Sham_ = 0.728), which was more accurate than the Cathodal group. We did not find a three-way interaction of Difficulty Level, Conflict Level, and Stimulation; a two-way interaction of Difficulty Level and Stimulation; or a main effect of Stimulation for any contrasts.

#### Garden-path reading time

We examined the real-time processing effects of garden-path recovery by measuring button presses to individual words as participants read sentences. We removed trials with incorrect responses to comprehension questions (loss of 16.7% of trials) in order to examine cases when garden-path recovery was successful, or when readers correctly revised an initial misinterpretation. Of the correct trials, we then removed button press times that were less than 200ms and greater than 2000ms (loss of 3.04% of words). The remaining raw word-by-word button presses were summed to create four *a priori* regions of interest (as delimited by slashes in Table 1). The final region of ambiguous sentences contains the disambiguating information (“sparkled brightly”), which is where we would expect to find any effects related reanalysis—namely, those mediated by executive-control [107–108]. We present mixed-models only for reading times in this final region. Note that due to the reversed clause order of control sentences, this region contains different content in the unambiguous items (e.g., “hid”). We justified using the sentence-final region as our comparison to control for “wrap-up effects” (see also [28]). Finally, because sentence regions often varied in string length (and length is a highly reliable predictor of sentence reading time), all mixed-models also included a covariate of region string length in terms of number of characters (see [109] for additional rationale).

**Fig 4:**
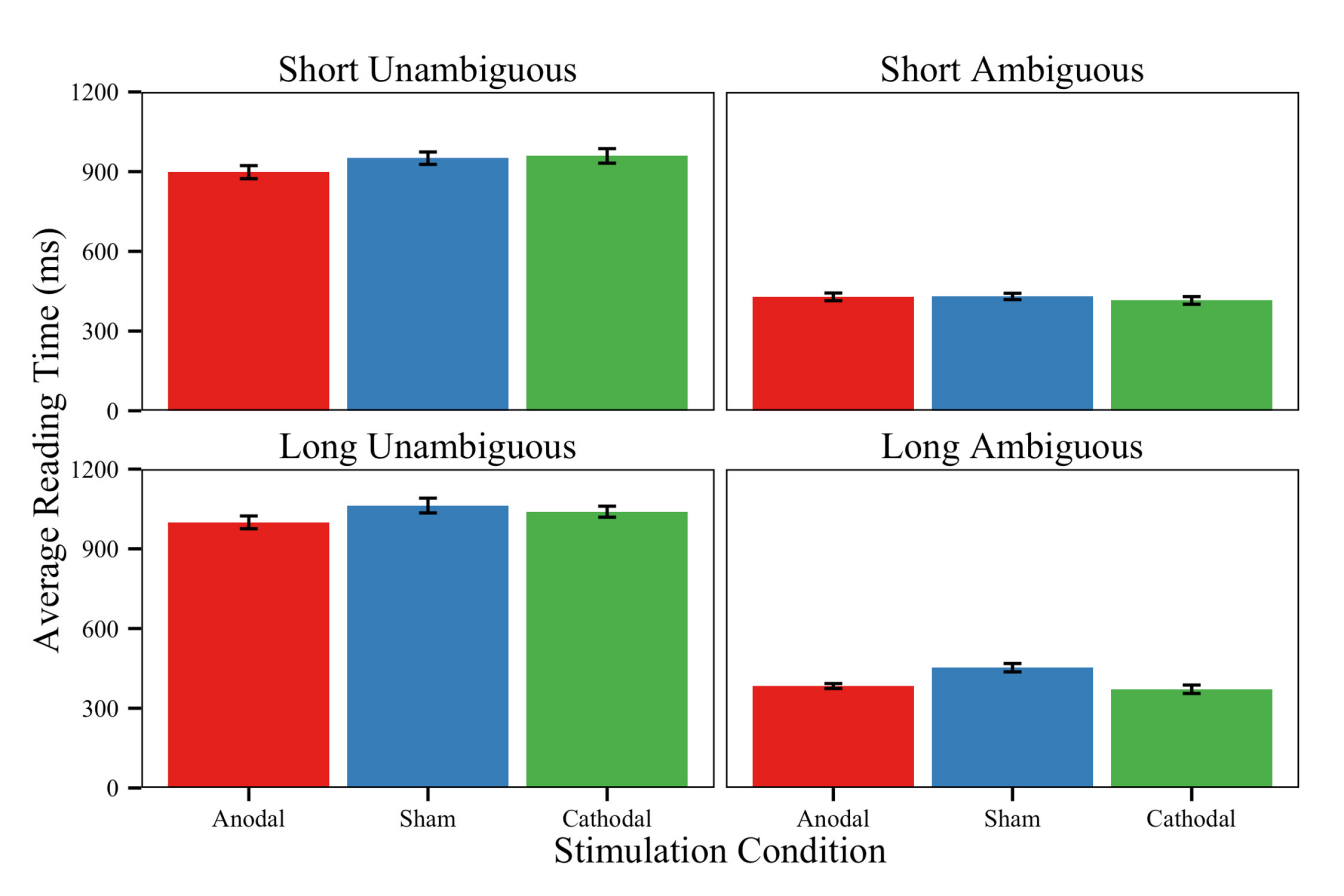
Garden-path reading times. Average uncorrected reading times on the garden-path sentence conditions in the region of interest (Note: the content of this region varied for sentence type, such that it was “sparkled brightly” in ambiguous sentences and “hid” in unambiguous sentences). Reading times are plotted for correct items only for each stimulation group and task condition. Error bars = ±1 standard error of the mean.

Table 3 includes the model estimates of reading times in Region 4. Although there was no main effect of Difficulty Level, we observed a significant main effect of Conflict Level, alongside an interaction between Conflict Level and Difficulty Level. Ambiguous final regions were read more slowly than unambiguous final regions (M_AM_ = 1036ms vs. M_UN_ = 411ms, uncorrected; see Fig 4). Moreover, the conflict effect (Ambiguous–Unambiguous performance) was larger for Long sentences compared to short ones (M_Long_ = 666 ms vs. M_Short_ = 558ms, uncorrected).

**Table 3:** Estimate coefficients from linear mixed-effects models for reading times in the critical area of garden-path sentences.

Predictor	Coefficient	SE	t-value
Fixed Effects			
**Intercept**	**375.654**	**47.873**	**7.847** *
**Region Length (covariate)**	**42.886**	**3.609**	**11.885** *
**Conflict Level**	**-270.012**	**43.070**	**-6.269** *
Difficulty Level	5.203	26.337	0.198
**Conflict Level x Difficulty Level**	**-109.195**	**53.975**	**-2.023** *
Anodal vs. Cathodal	41.874	47.571	0.880
Anodal vs. Sham	22.178	47.581	0.466
Sham vs. Cathodal	19.291	48.021	0.402
Conflict Level x Anodal/Cathodal	-67.973	41.556	-1.636
Conflict Level x Anodal/Sham	-17.775	41.518	-0.428
Conflict Level x Sham/Cathodal	-50.207	42.953	-1.169
Difficulty Level x Anodal/Cathodal	17.106	26.400	0.648
**Difficulty Level x Anodal/Sham**	**56.049**	**26.386**	**2.124** *
Difficulty Level x Sham/Cathodal	-38.680	27.211	-1.421
Conflict Level x Difficulty Level x Anodal/Cathodal	-31.638	57.410	-0.551
Conflict Level x Difficulty Level x Anodal/Sham	-76.467	57.299	-1.335
Conflict Level x Difficulty Level x Sham/Cathodal	42.734	58.987	0.470
Random Effects			
Subjects (Intercept)	27693		
Subjects (Conflict Level)	13706		
Subjects (Difficulty Level)	156		
Subjects (Conflict Level x Difficulty Level)	6830		
Items (Intercept)	9471		
Items (Conflict Level)	30988		
Items (Difficulty Level)	16355		
Items (Conflict Level x Difficulty Level)	61123		
Residuals	89428		

The critical region (Region 4) is “sparkled brightly” in ambiguous sentences and “hid” in unambiguous sentences. Difficulty Level refers to the length of the sentence (long vs. short) and Conflict Level refers to sentence ambiguity (ambiguous vs. unambiguous). Bold indicates coefficients that are significant. SE = standard error

*significant at the p<0.05 level

Although there was no main effect of Stimulation, we observed an interaction of Difficulty Level and Stimulation for the Anodal/Sham contrast, such that the Anodal group was faster to read long sentences compared to the Sham group (M_Anodal_ = 793ms vs. M_Sham_ = 870ms, uncorrected). The Cathodal group did not differ from any other group when reading long sentences but was numerically slower than the Sham group, as expected (M_Cathodal_ = 901ms). No effects appeared for the remaining sentence regions (*t*s<0.91, *p*s>0.36). We also did not observe any interactions of Conflict Level and Stimulation or any interactions of Difficulty Level, Conflict Level, and Stimulation for the contrasts.

Considering both measures of garden-path recovery, we provide initial evidence that acute brain stimulation to left LPFC can influence sentence processing in real-time reanalysis and offline comprehension accuracy. Left LPFC seems to service both conflict and difficulty demands within garden-path recovery. Sentence reading time measures show positive effects of Anodal stimulation in the sentence-final region for only long sentences, regardless of syntactic ambiguity. Later comprehension accuracy is best for the Anodal stimulation group on only ambiguous sentences with heightened executive-control demands, regardless of sentence length.

#### Relative clause reading time

In addition to garden-path sentences, participants read relative clause sentences. These items were included as controls to examine the real-time processing effects on constructions containing linguistic complexities linked to other general-purpose cognitive abilities aside from executive-control (i.e., working memory, linguistic integration, and retrieval demands). We predicted no effects in the critical embedded clause region of these items if stimulation of left LPFC selectively influences executive-control. If the Difficulty Level-by-Stimulation effects reported above for garden-path reading times were due to task complexity alone, then other cases of complexity should show comparable results. Namely, we would expect to also see effects of relative clause Sentence Type (object-extracted are more demanding the subject-extracted clauses) and Sentence Length (longer sentences should be more difficult to process compared to shorter items).

**Fig 5:**
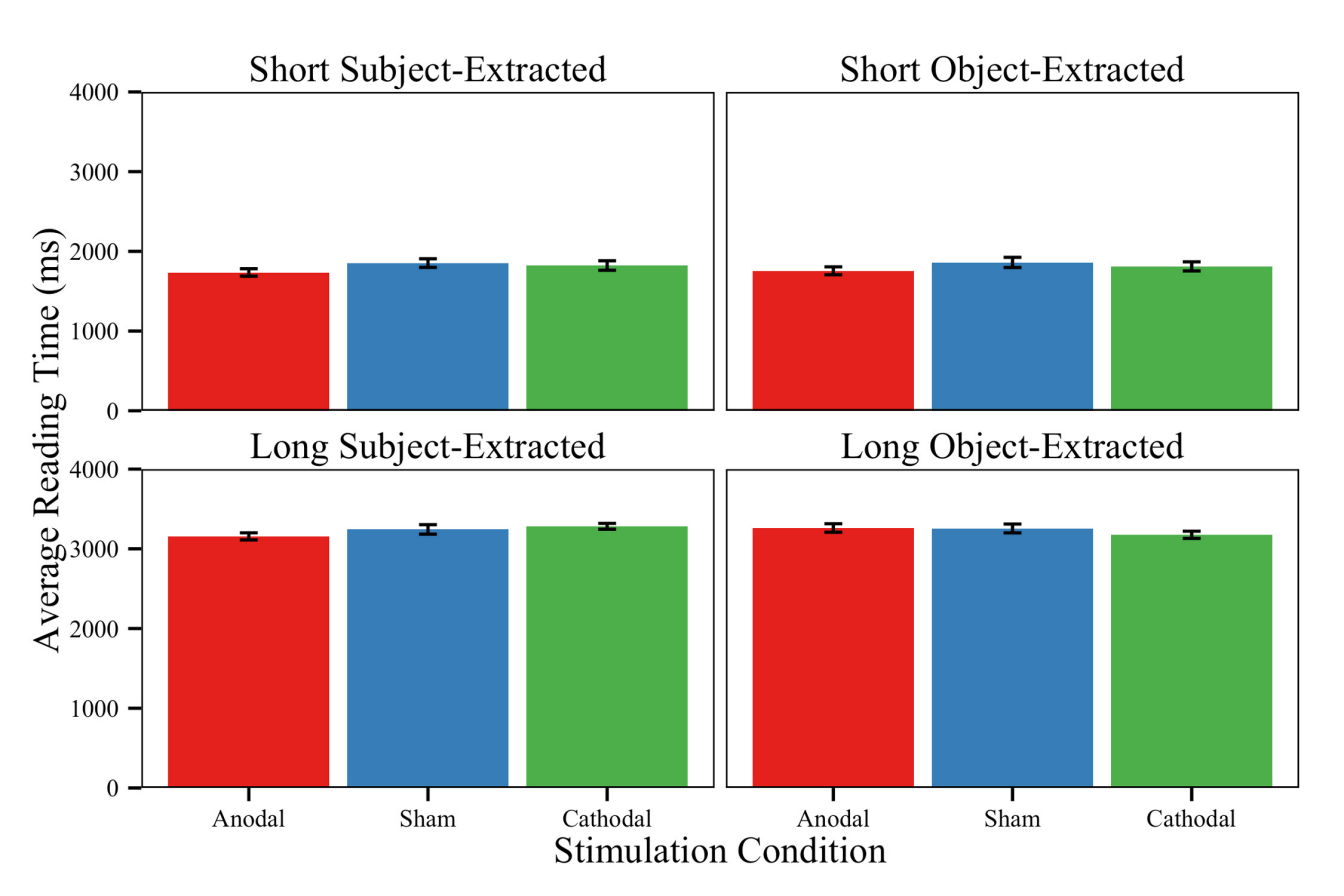
Relative clause reading times. Average uncorrected reading times on the relative clause sentence conditions in the region of interest (Note: the content of this region varied for sentence type, such that it was “who the expert questioned” in object-extract sentences and “who questioned the expert” in subject-extracted sentences). Reading times are plotted for correct items only for each stimulation group and task condition. Error bars = ±1 standard error of the mean.

We used a similar data processing pipeline from the garden-path sentences for the relative clause reading times. Incorrect trials were removed (loss of 16.5% of trials), as well as button press times that were less than 200ms and greater than 2000ms (loss of 3.65% of words). The remaining word-level button presses were chunked into four regions of interest (see Table 1), with the embedded clause (Region 2) being the critical sentence area. The mixed models of reading times in this critical region included a covariate of Region Length to control for number of characters. In terms of task-level effects, we observed only a main effect of Difficulty Level, such that longer sentences were read more slowly than short sentences (M_Long_ = 3253ms vs. M_Short_ = 1814ms, uncorrected). The model revealed no effects of Sentence Type and no effects involving Stimulation for any contrasts (Table 4; Fig 5). The lack of any reliable interactions containing Stimulation suggests that active stimulation to left LPFC does not have an effect on difficult sentence processing conditions broadly construed (i.e., when executive-control demands are removed).

**Table 4:** Estimate coefficients from linear mixed-effects models for length-corrected reading times in the critical region of relative clauses.

Predictor	Coefficient	SE	t-value
Fixed Effects			
**Intercept**	**2110.047**	**239.210**	**8.821** *
**Region Length (covariate)**	**23.848**	**5.976**	**3.991** *
Sentence Type	-60.559	59.142	-1.024
**Difficulty Level**	**1561.640**	**181.100**	**8.623** *
Sentence Type x Difficulty Level	-10.250	114.292	-0.090
Anodal vs. Cathodal	111.545	197.297	0.565
Anodal vs. Sham	119.027	197.283	0.603
Sham vs. Cathodal	-7.482	199.158	-0.038
Sentence Type x Anodal/Cathodal	-0.016	84.547	0.000
Sentence Type x Anodal/Sham	130.423	84.421	1.545
Sentence Type x Sham/Cathodal	-130.440	85.393	-1.528
Difficulty Level x Anodal/Cathodal	156.660	158.414	0.989
Difficulty Level x Anodal/Sham	99.593	158.272	0.629
Difficulty Level x Sham/Cathodal	57.067	159.889	0.357
Sentence Type x Difficulty Level x Anodal/Cathodal	-23.375	163.493	-0.143
Sentence Type x Difficulty Level x Anodal/Sham	52.945	163.158	0.324
Sentence Type x Difficulty Level x Sham/Cathodal	-76.319	165.118	-0.462
Random Effects			
Subjects (Intercept)	499255		
Subjects (Difficulty Level)	267024		
Subjects (Sentence Type)	29515		
Subjects (Sentence Type x Difficulty Level)	93252		
Items (Intercept)	8639		
Items (Difficulty Level)	14575		
Residuals	634184		

Region 2 is the critical sentence region. For relative clauses, Difficulty Level refers to the length of the embedded clause region (long vs. short) and Sentence Type refers to extraction (object-extracted vs. subject-extracted). Bold indicates coefficients that are significant. SE = standard error

*significant at the p<0.05 level

### *N*-Back Performance

We evaluated the effects of left LPFC stimulation on recognition memory with a modified *n*-back task that parametrically introduced conflict (high: *n*-back-with-lures; low: *n*-back-without-lures) and difficulty demands (high: 4-back; low: 2-back). Performance was assessed with non-parametric signal detection indices of target/non-target discrimination (A′) and response criterion (Grier’s B′′ [110]). Measures were computed separately for each subject for each of the 4 task conditions.

#### Discrimination (A′)

**Fig 6:**
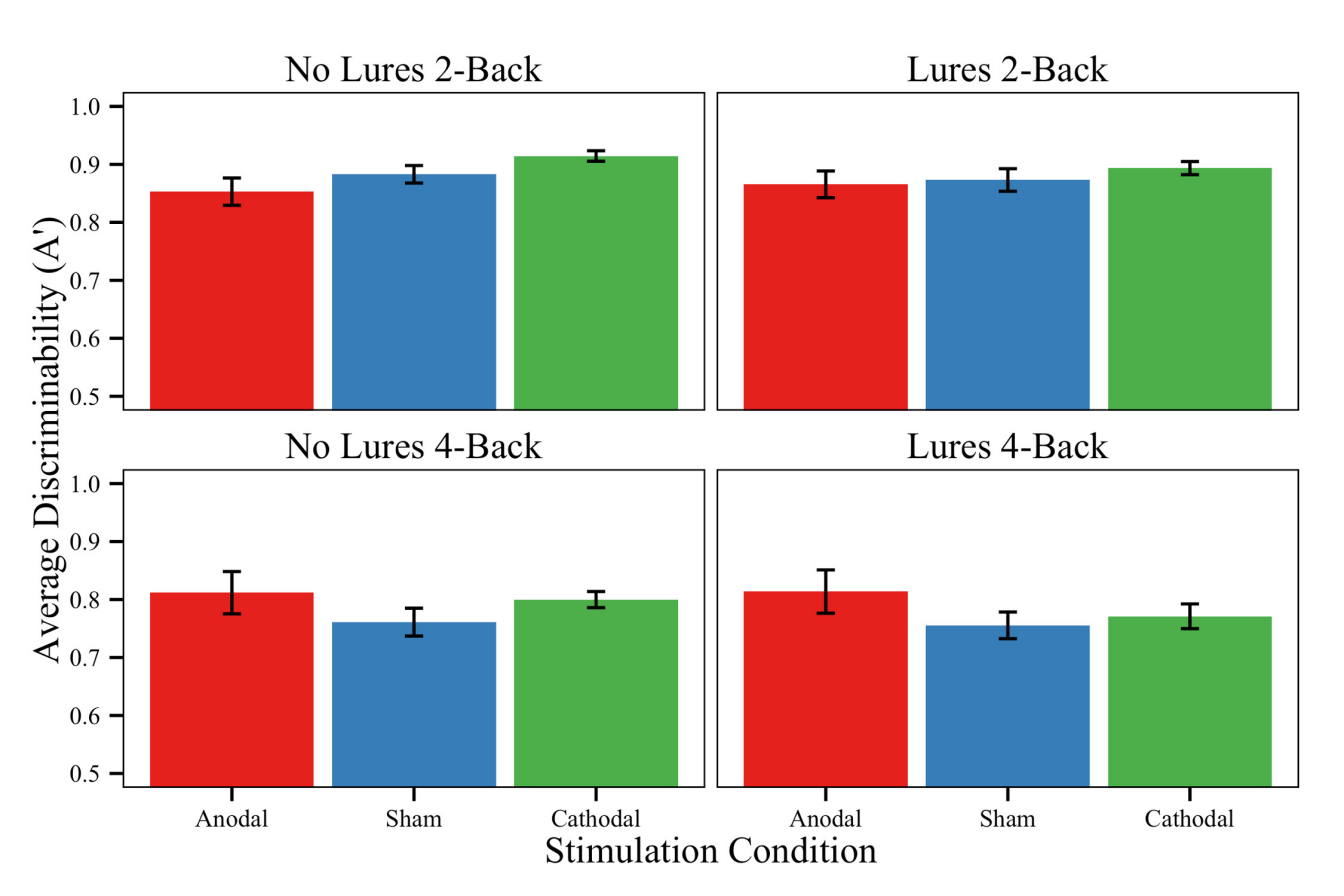
*N*-back discriminability. Average *n*-back discriminability (A′) for each stimulation group and task condition. A′ is a non-parametric signal detection index of how well participants can discriminate targets from non-targets; thus a higher A′ value corresponds to better discriminability. Error bars = ±1 standard error of the mean.

In terms of task-level effects, the model of A′ did not reveal a significant interaction between Difficulty Level and Conflict Level or a main effect of Conflict Level. We did, however, observe a *marginal* main effect of Difficulty Level, such that all participants were numerically better able to discriminate targets from non-targets on 2-back blocks compared to 4-back blocks (M_2Back_ = 0.880 vs. M_4Back_ = 0.786; Table 5 and Fig 6).

**Table 5:** Estimate coefficients from linear mixed-effects models for *n*-back discriminability (A′).

Predictor	Coefficient	SE	t-value
Fixed Effects			
**Intercept**	**0.837**	**0.017**	**48.507** *
Conflict Level	-0.008	0.006	-1.179
*Difficulty Level*	*0*.*047*	*0*.*024*	*1*.*941* ^†^
Conflict Level x Difficulty Level	-0.010	0.013	-0.806
Anodal vs. Cathodal	0.005	0.025	0.217
Anodal vs. Sham	-0.020	0.025	-0.800
Sham vs. Cathodal	0.025	0.025	1.006
**Conflict Level x Anodal/Cathodal**	**0.032**	**0.009**	**3.545** *
Conflict Level x Anodal/Sham	0.016	0.009	1.689
Conflict Level x Sham/Cathodal	0.017	0.009	1.849
**Difficulty Level x Anodal/Cathodal**	**0.072**	**0.035**	**2.082** *
**Difficulty Level x Anodal/Sham**	**0.072**	**0.035**	**2.066** *
Difficulty Level x Sham/Cathodal	0.001	0.035	0.007
Conflict Level x Difficulty Level x Anodal/Cathodal	0.002	0.018	0.136
Conflict Level x Difficulty Level x Anodal/Sham	0.015	0.018	0.826
Conflict Level x Difficulty Level x Sham/Cathodal	-0.012	0.019	-0.660
Random Effects			
Subjects (Intercept)	0.0078		
Subjects (Difficulty Level)	0.0148		
Residuals	0.0026		

Difficulty Level refers to the *n*-level contrast of 2-back and 4-back, and Conflict Level refers to the contrast of blocks with versus without lures. Bold indicates coefficients that are significant. SE = standard error

*significant at the p<0.05 level

^†^marginal at the p<0.06 level

With regard to stimulation effects, we found a reliable interaction of Difficulty Level and Stimulation. The Anodal group outperformed the Cathodal and Sham groups in terms of discriminability on more difficult *n*-back sequences (4-back), regardless of the presence of interference lures. Indeed, the Anodal group demonstrated better discriminability (i.e., larger A′ values) relative to the remaining groups on the 4-back blocks (M_Anodal_ = 0.813, M_Cathodal_ = 0.785, M_Sham_ = 0.758), but not on the 2-back blocks (M_Anodal_ = 0.859, M_Cathodal_ = 0.904, M_Sham_ = 0.878). The difference between the Sham and Cathodal did not reach significance. In addition, the model revealed a reliable interaction of Conflict Level and Stimulation, such that the Anodal group outperformed the Cathodal group in terms of target/non-target discrimination on *n*-back blocks with lures (M_Anodal_ = 0.840, M_Cathodal_ = 0.832), but that the opposite was true for *n*-back blocks *without* lures (M_Anodal_ = 0.832, M_Cathodal_ = 0.857). Put differently, the Cathodal group shows more of a decline in performance for blocks with lures, a deficit that active stimulation over left LPFC may have protected subjects against in the Anodal group. Despite this, the model did not show the same interaction for the anodal/sham contrast. We also did not find a main effect of Stimulation or a reliable 3-way interaction of Difficulty Level, Conflict Level, and Stimulation for any of the group comparisons.

#### Response criterion (Grier’s B′′)

**Fig 7:**
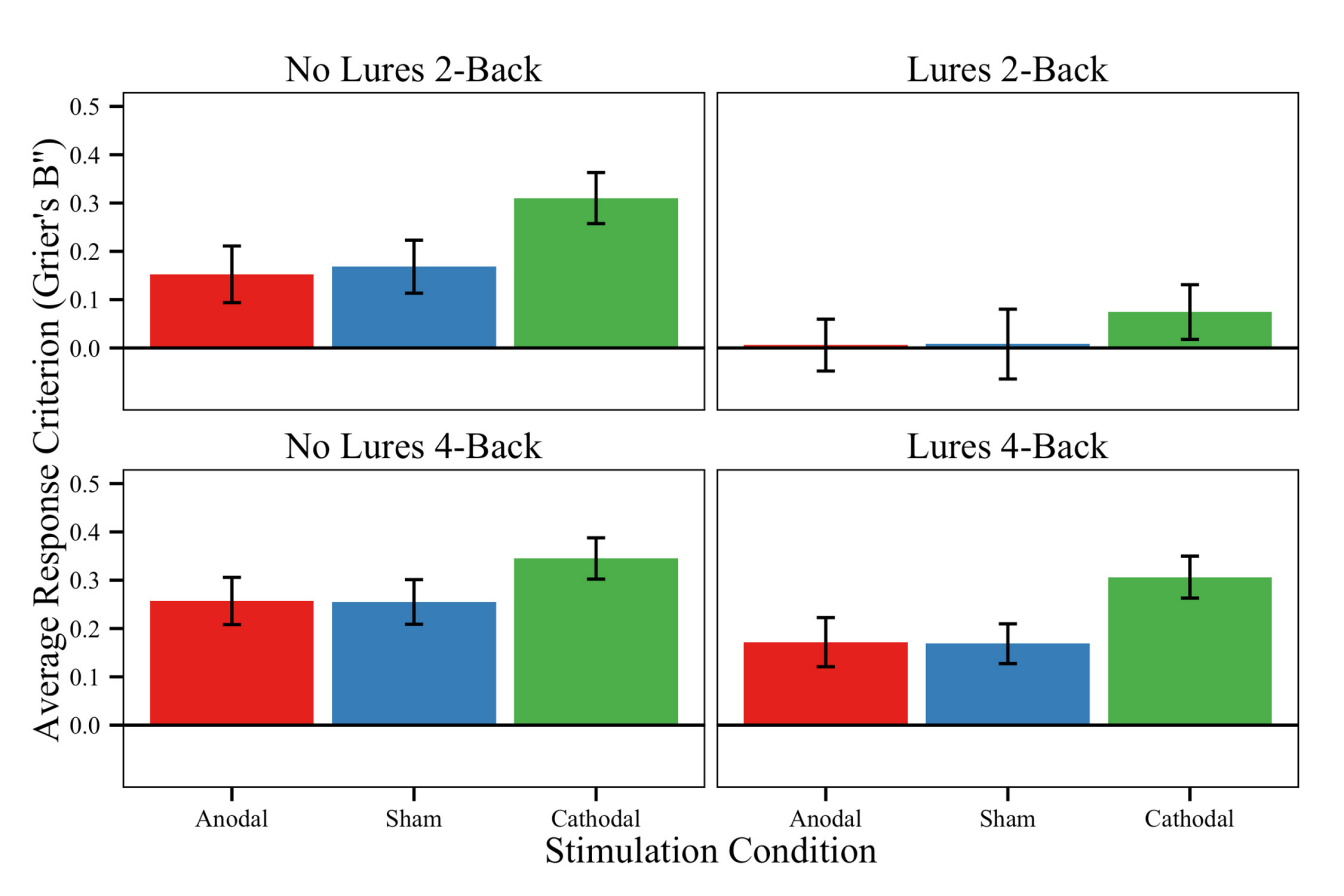
*N*-back response thresholds. Average *n*-back response criterion (Grier’s B′′) for each stimulation group and task condition. Grier’s B′′ is a non-parametric signal detection index of participants’ bias to respond ‘target or ‘non-target.’ A higher Grier’s B′′ value corresponds to a conservative bias to say ‘non-target,’ while a lower Grier’s B′′ indexes a higher likelihood of judging an item to be a ‘target.’ Error bars = ±1 standard error of the mean.

The mixed-effects model of Grier’s B′′ resulted in several task-level effects. Specifically, we observed significant main effects of Difficulty Level, Conflict Level, and an interaction of these two factors (Table 6). Participants were more conservative on 4-back relative to 2-back blocks (M_4Back_ = 0.251 vs. M_2Back_ = 0.132) and on *n*-back-without-lures compared to cases with lures (M_NoLures_ = 0.254 vs. M_Lures_ = 0.129; see Fig 7). Interestingly, the interaction between Difficulty Level and Conflict Level was driven by the lowest (least conservative) response criterion on the 2-back-with-lures block (M = 0.042 vs. all other M’s>0.216). Perhaps, this is because the 2-back-with-lures block was participants’ first exposure to lure items, which may have resulted in more ‘yes’ responses than normal.

**Table 6:** Estimate coefficients from linear mixed-effects models for *n*-back response criterion (Grier’s B′′).

Predictor	Coefficient	SE	t-value
Fixed Effects			
**Intercept**	**0.138**	**0.035**	**3.977** *
**Conflict Level**	**0.119**	**0.019**	**6.095** *
**Difficulty Level**	**-0.147**	**0.050**	**-2.949** *
**Conflict Level x Difficulty Level**	**0.085**	**0.039**	**2.189** *
**Anodal vs. Cathodal**	**0.118**	**0.050**	**2.377** *
Anodal vs. Sham	0.009	0.050	0.177
**Sham vs. Cathodal**	**0.107**	**0.051**	**2.101** *
Conflict Level x Anodal/Cathodal	0.023	0.028	0.839
Conflict Level x Anodal/Sham	-0.010	0.028	-0.355
Conflict Level x Sham/Cathodal	0.014	0.029	0.479
Difficulty Level x Anodal/Cathodal	0.015	0.071	0.208
Difficulty Level x Anodal/Sham	0.034	0.072	0.477
Difficulty Level x Sham/Cathodal	-0.022	0.077	-0.291
**Conflict Level x Difficulty Level x Anodal/Cathodal**	**0.122**	**0.055**	**2.200** *
Conflict Level x Difficulty Level x Anodal/Sham	-0.041	0.056	-0.729
**Conflict Level x Difficulty Level x Sham/Cathodal**	**0.124**	**0.058**	**2.140** *
Random Effects			
Subjects (Intercept)	0.0301		
Subjects (Difficulty Level)	0.0573		
Residuals	0.0241		

Difficulty Level refers to the *n*-level contrast of 2-back and 4-back, and Conflict Level refers to the lure presence contrast of blocks with versus without lures. Bold indicates coefficients that are significant. SE = standard error

*significant at the p<0.05 level

Considering stimulation-based effects, we found a significant *three-way* interaction of Difficulty Level, Conflict Level, and Stimulation for the cathodal/sham and cathodal/anodal contrasts. These interactions are due to large differences between the Cathodal group and the remaining groups on 2-back-without-lures and 4-back-with-lures (difference of 0.176 vs. 0.135, respectively), but not on 2-back-with-lures and 4-back-without-lures (difference of 0.091 vs. 0.088). Despite this, we found no two-way interactions of Conflict Level and Stimulation or Difficulty Level and Stimulation for any contrasts. We did, however, note a significant main effect of Stimulation group, indicating that the Cathodal group demonstrated a higher likelihood of responding ‘non-target’ (i.e., higher Grier’s B′′ values, or a more conservative response criterion) compared to the Anodal and Sham groups, in general (M_Anodal_ = 0.147, M_Sham_ = 0.151; M_Cathodal_ = 0.259).

These *n*-back results indicate that Anodal stimulation over left LPFC gives rise to performance boosts in terms of *n*-back discriminability on cases of heightened conflict and difficulty (i.e., both when lures are introduced and when *n*-level is high), but not in terms of response criterion.

## Discussion

**Fig 8:**
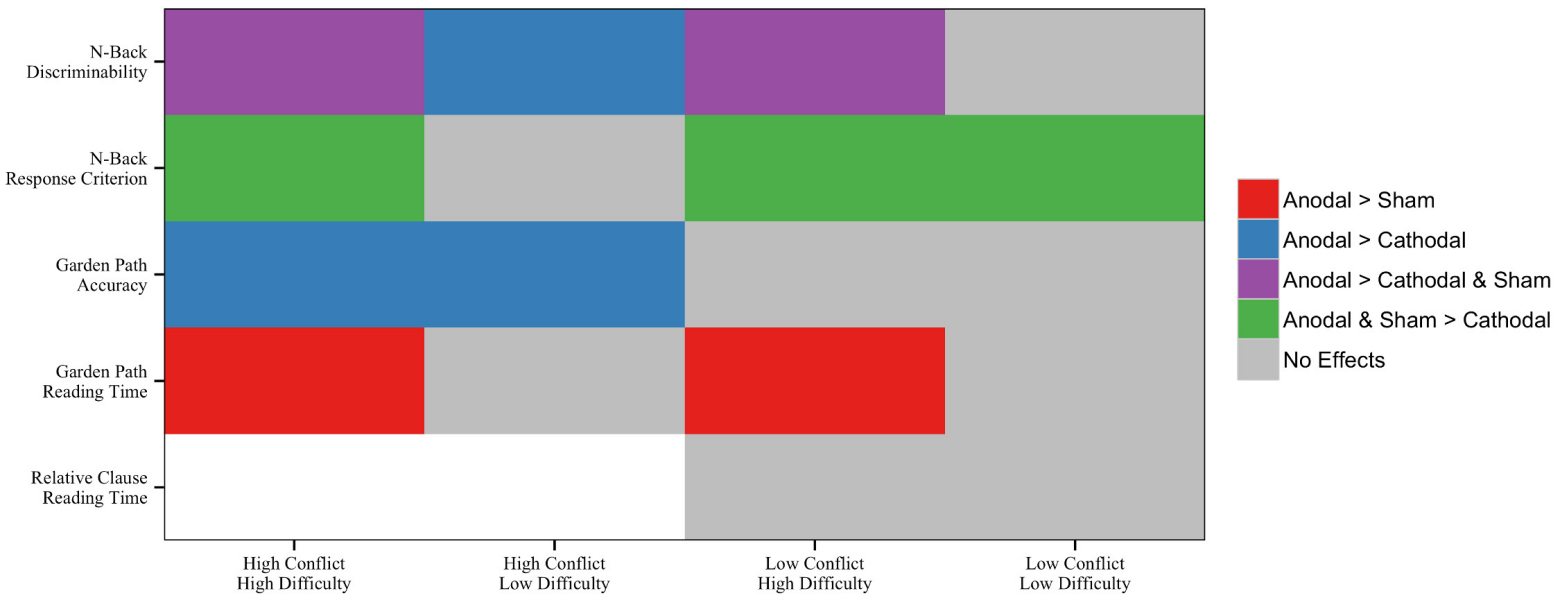
Summary of results. Interactions of Conflict Level and Difficulty Level with Stimulation in the *N*-Back and Reading Tasks. Note that the relative clause items have a no Conflict Level effects to evaluate. Colors indicate cases when the Stimulation contrasts significantly predicted performance. Gray shading = no effects for any contrast; Red shading = Anodal > Sham; Blue shading = Anodal > Cathodal; Purple shading = Anodal outperforms remaining groups; Green shading = Anodal and Sham outperform Cathodal.

We demonstrated that participants who received Anodal stimulation of left LPFC outperformed those assigned to receive Cathodal or Sham stimulation on most task conditions requiring executive-control in the memory and language domains. Fig 8 summarizes our results. Colored panels, which tag cases when the Anodal group outperformed the remaining groups in some way, appear primarily on the left half of the figure where executive-control demands are high (i.e., “high conflict” cases). This pattern generally holds, with the exception of four instances on the right half of the figure; these represent cases when heightened task difficulty improved as a function of Anodal stimulation. Taken together, these results suggest that Anodal stimulation of left LPFC is associated with consistent benefits for cases tapping executive-control. We also note a similar pattern for difficult task conditions that do not involve resolving interference.

To recap, on the *n*-back task, we observed an effect of tDCS for target/non-target discriminability, such that the Anodal stimulation group was more sensitive to detect targets at a higher *n*-level relative to Cathodal and Sham controls, while the Cathodal and Sham groups performed no differently from one another. This is well aligned with results of a recent meta-analysis that reported more prominent effects of anodal stimulation for higher working memory loads (i.e., at higher *n*-levels) [58]. In addition to this, our findings provide two new contributions to the tDCS literature. First, compared to the Cathodal group, the Anodal group exhibited superior discriminability on *n*-back versions with lures, but not on *n*-back-without-lures. In particular, the Cathodal group shows a decline in discriminability on lure trials relative to those without lures, while the Anodal group is equally good at discriminating targets from non-targets regardless of the presence of lures. This is a result we expected given the mounting functional imaging evidence for the role of left LPFC for *n*-back interference lure processing [60,111–112]. Second, the Cathodal group was more conservative than the remaining groups, indicating that cathodal stimulation prompted more conservative responding compared to anodal and sham stimulation; this pattern is consistent with at least one other report demonstrating that anodal stimulation gives rise to less conservative *n*-back response thresholds [113]. Another plausible explanation for these data is that participants receiving cathodal stimulation may have engaged in a compensatory task-specific strategy to avoid false alarming to non-targets. Additional work should systematically explore the replicability of this cathode-based impairment on the *n*-back task. Finally, because *n*-back was always performed after the reading task, it is possible that the effects we observed were due, in part, to the limited amount of simultaneous (“online”) stimulation. That is, much of the *n*-back task was performed after the 30-minute stimulation period concluded. Even so, prior work indicates that anodal stimulation of left LPFC improves *n*-back accuracy for up to 30 minutes following stimulation [57]. Although much work demonstrates convincing online *and* offline effects [114–115], only some attempts have been made to understand the temporal properties of stimulation [32], which may be an important source of variability that contributed to our findings and that future studies should explore.

On the sentence reading task, we observed several novel results favoring the Anodal group. First, compared to cathodal and sham, anodal stimulation resulted in higher accuracy on comprehension questions following ambiguous garden-path sentences, but not unambiguous control sentences. Although this result corroborates the discriminatory role of left LPFC for sentence processing scenarios with heightened executive-control demands [8], the reading time data paint a slightly different picture. We observed a positive effect of anodal stimulation for difficult ambiguous and unambiguous sentences (i.e., those with an additional modifier phrase). That is, the benefits of left LPFC stimulation were not limited exclusively to real time processing decisions involving executive-control. Interestingly, further assessment of this analogous factor in relative clause constructions did not reveal any effects of stimulation. The difficult sentence type (object-extracted clause) and all long sentences were read equally quickly regardless of stimulation group assignment, suggesting that left LPFC may not service all linguistically complex input, but rather supports certain cases of real-time sentence processing [20]. Interestingly, cathodal and sham stimulation did not differ in their performance profiles for garden-path recovery or relative clause parsing, providing some evidence that cathodal stimulation does not result in behavioral changes as strong as those routinely observed with anodal stimulation [116–117].

### Difficulty versus Executive-Control Demands

The distinction that we make between conditions with heightened executive-control demands versus those with elevated difficulty due to factors other than information-conflict is one that has been fleshed out elsewhere. This distinction has been articulated in terms of interference-resolution versus working memory maintenance [118–119], non-mnemonic versus mnemonic processing [27,120–121], and functional versus architectural abilities [122]. Thus, there is some impetus to disentangle these processing demands when considering the unique role of left LPFC for executive-control. We argue that our findings offer one such attempt to assess the unique role of executive-control (separate from task-difficulty) in higher-level cognitive domains like language.

Related work suggests that general-purpose executive-control and processing associated with domain-specific difficulty are supported by neighboring neural areas within left LPFC [22,123]. This opens up another potential explanation for our mixed results. Namely, we observed that some Stimulation effects interacted with Conflict-Level *only* while others hinged on Difficulty-Level. Perhaps the adjacent anatomical nature of regions reputed to support executive-control and task-specific difficulty means that tDCS led to changes in conditions with both processing demands. Although we used “high definition” electrodes to administer the current, the technique is still lacking the necessary focal imprecision to discriminate among nearby regions. Ideally, in future work, anatomical variability across participants should be accounted for by identifying anode and cathode locations on the basis of individualized structural and functional information [52,124–125]. Moreover, high-definition montages arranged in a 4x1 ring can be combined with subject-by-subject anatomical information to provide the cleanest avenue to target neural tissue functionally responsive to conditions tapping executive-control versus task-specific difficulty [126–127].

Our current design relies on proper comparisons of minimally different conditions to parametrically introduce executive-control demands and task difficulty; however, it is possible that our results are still driven by an amalgam of cognitive mechanisms that we have not considered or accounted for here. For instance, the electrode sites used in the present study tap executive control (F3) *and* visual processing (O1), bringing to the forefront the possibility that impairing one ability while boosting the other might offset the intended effects of stimulation in important ways [128]. Given that the current reading and *n*-back tasks rely on visual processing, the lack of cathodal findings here could be, in part, due to the dynamic interplay of these two cognitive systems. It is possible that the positive effects of anodal stimulation over one region could overwhelm any simultaneous cathodal effects over another region. Future work might probe this possibility by testing the effects of electrode placement within the context of a multifocal stimulation approach [36].

It is also possible that the present design could benefit from fine-grained outcome measures, including functional imaging, connectivity indices, or eye movements. The lattermost option would remove some demands associated with our current reading task; it involved a self-paced moving-window design, which prevents naturalistic text processing and places a premium on working memory resources such that later arriving words obliterate prior ones [95,129]. Finally, the strongest tDCS reports incorporate some level of computational modeling to identify unique mechanistic underpinnings [130]. Some combination of the abovementioned improvements is liable to create the best scenario for interpreting stimulation-mediated changes in behavior.

### Brain Stimulation Considerations

To evaluate the effects of Anodal stimulation, our design included two comparison groups (Cathodal and Sham). Fig 8 depicts conditions when Anodal stimulation leads to better performance relative to Sham stimulation (denoted in red), Cathodal stimulation (denoted in green), or both control conditions (denoted in blue). Although Anodal stimulation typically improves performance, it does so with respect to different comparison groups. Generally, the Anodal group outperforms the Cathodal group on all measures except for reading time on long garden-path sentences. The Sham group, on the other hand, only underperforms relative to the Anodal group on high-difficulty cases as measured by 4-back discriminability and reading time on long garden-path sentences. The lack of a difference between the Anodal and Sham groups for comprehension accuracy is trending in the anticipated direction, such that the Anodal group is numerically more accurate than the Sham group on questions following ambiguous sentences (see right panels of Fig 3). Interestingly, when we compare performance between the Cathodal and Sham groups, we only find effects for *n*-back response criterion, such that the Cathodal group is more conservative than the remaining stimulation groups. No other measures revealed a distinction between the Cathodal and Sham groups. This pattern is contrary to some researchers’ findings [34,114,131], though consistent with others’ [116–117]. Take together, the current study provides some evidence for the efficacy of cathodal stimulation as an active control for anodal stimulation under some situations.

Brain stimulation offers an acute approach to evaluate brain-behavior relationships, as well as the linking hypotheses governing these relationships; however, there are several methodological limitations of tDCS worth considering. As touched on in the previous section, more precise electrode placement is a prerequisite to ensure that the same brain region is targeted across individuals. Our approach used canonical neuroanatomical markers (i.e., prescribed distances from the inion and nasion) to place the electrodes, but MRI-supported electrode arrangements offer an alternative to mitigate some uncertainty about which regions are receiving current ([132], but see [133] for arguments on the consistency across brains). Nevertheless, stimulation is still not necessarily focalized to the brain regions directly under the anode [115,134–135]. This may be due to reversals in polarity that occur across cerebral folds near the electrode site, such that the electrode montage does not always dictate consistent current flow [130]. Modeling the current on an individual subject basis by relying on structural and functional scans may help to understand and minimize this issue. A second limitation of tDCS is that almost all higher-level cognitive tasks, including executive-control, are supported by large-scale functional networks that include areas other than those routinely targeted by stimulation techniques [136–138]. Fortunately, initial evidence indicates that tDCS leads to changes on a network-wide level [139], yet the question of focality and precision of stimulation remains [39]. Exciting new research suggests that we may be able to rely on graph theory to target “network hubs” to elicit the largest network-level effects [140]. A final concern stems from a recent observation that not every person responds to tDCS stimulation. This may be due to variability in neuronal orientation or to other factors described above [141]. One way to combat this is to collect data from a sufficiently large sample, as we have done here.

### Implications

We sidestep some of the methodological limitations common to tDCS by leveraging our interpretations on 1) our design with minimal within-task control conditions and 2) converging evidence from other methods and populations that hypothesize a clear role of executive-control in certain memory and language task conditions. The current findings provide additional evidence for left LPFC as the mediating force behind the linking hypothesis involving executive-control for garden-path recovery. In addition to these theoretical implications, the current results may be harnessed into applications for special populations. Specifically, we believe that these findings may be used to guide the development of interventions for patients with focal insult to left LPFC. Indeed, the number of successful attempts to use tDCS to improve abilities in individuals with aphasia and stroke patients with language-specific impairments is growing [65,67,142–146].

The present results may also have implications for healthy individuals. In particular, intervention-based training is a complementary tool that may also temporarily enhance higher-level cognitive abilities, like language and memory performance. Some groups have begun combining both methods and have found mixed results [115,147–151]. It is possible that with appropriate linking hypotheses between training and transfer measures, joint tDCS/cognitive training interventions may give rise to improvements in sentence processing under difficult conditions, replicating prior cognitive training findings in this area [28]. Regardless, our results offer some promise of using tDCS in a single-session to determine appropriate training/transfer task combinations [27], and importantly, they add to the mounting evidence for the selective role of left LPFC-mediated executive-control for tasks in the language domain.

## Supporting Information

S1 Table Sentence stimuli used in the reading task. All critical sentence versions used in the experiment for the garden-path and relative clause constructions.
(PDF)

S1 File Participant data. Compressed file containing data files from the *N*-Back Task (response times and accuracy for each trial) and Reading Task (word-by-word button press times and comprehension accuracy for each trial), as well as a separate file with participant information including group assignment and demographic information.
(ZIP)
