# Supplementary material for: Language and Memory Improvements following tDCS of Left Lateral Prefrontal Cortex
Source: PLoS One. 2015 Nov 3;10(11):e0141417. doi: 10.1371/journal.pone.0141417 (PMC4631603; doi:10.1371/journal.pone.0141417)
Supplement: S1 Table — All critical sentence versions used in the experiment for the garden-path and relative clause constructions. (PDF) [file pone.0141417.s001.pdf]

Sentence Type	Stimulus Sentence	Comprehension Question
Garden-Path	As Chris worked out the issue (that was confusing and unclear) continued to worsen.	Did Chris work out?
Garden-Path	While Kristin put make-up on the model (that was tall and thin) put on her outfit.	Did Kristin put make-up on?
Garden-Path	As the artist put make-up on the actress (that was famous and beautiful) walked onto the set.	Did the artist put make-up on?
Garden-Path	While the bride soaked the groom (that was handsome and smiling) put away his tuxedo.	Did the bride soak herself?
Garden-Path	As the dancer stripped the curtains (that were faded and musty) blocked the light.	Did the dancer strip?
Garden-Path	As the hero scratched the villain (that was sneaky and traitorous) kidnapped the blonde bombshell.	Did the hero scratch himself?
Garden-Path	As the maid changed the filter (that was old and dirty) collected dust.	Did the maid change?
Garden-Path	As the mother hid the cookies (that were warm and gooey) baked in the oven.	Did the mother hide?
Garden-Path	While the operator transferred the caller (that was eager and animated) accidentally hung up.	Did the operator transfer?
Garden-Path	As the secretary transferred the files (that were important and messy) collected on her desk.	Did the secretary transfer?
Garden-Path	While the tailor dressed the figurine (that was tall and shapely) fell over.	Did the tailor dress himself?
Garden-Path	While the woman disrobed the mannequin (that was frail and shapely) stood in the store.	Did the woman disrobe?
Garden-Path	As Betty woke up the neighbor (that was old and cranky) coughed loudly.	Did Betty wake up?
Garden-Path	While Jim bathed the child (that was blonde and pudgy) giggled with delight.	Did Jim bathe?
Garden-Path	As the adolescent washed the dishes (that were orange and greasy) sat in the sink.	Did the adolescent wash herself?
Garden-Path	While the boy washed the dog (that was white and furry) barked loudly.	Did the boy wash himself?
Garden-Path	As the chimps groomed the baboons (that were large and hairy) sat in the grass.	Did the chimps groom themselves?
Garden-Path	While the girl scratched the cat (that was gray and white) stared at the dog.	Did the girl scratch herself?

Sentence Type	Stimulus Sentence	Comprehension Question
Garden-Path	As the librarian lay down the book (that was intellectual and depressing) stayed on the shelf.	Did the librarian lie down?
Garden-Path	As the mother calmed down the children (that were tired and irritable) sat on the bed.	Did the mother calm down?
Garden-Path	As the nurse shaved the patient (that was tired and weak) watched TV.	Did the nurse shave?
Garden-Path	While the secretary calmed down the client (that was ruined and desperate) staked out the building.	Did the secretary calm down?
Garden-Path	As the student prepared the salad (that was healthy and fresh) remained in the refrigerator.	Did the student prepare herself?
Garden-Path	While the veterinarian showered the cat (that was sickly and thin) mewed in its cage.	Did the veterinarian take a shower?
Garden-Path	As Anna dressed the baby (that was small and cute) spit up on the bed.	Did Anna dress herself?
Garden-Path	While Frank dried off the car (that was red and shiny) sat in the driveway.	Did Frank dry himself off?
Garden-Path	While Robert changed the paint (that was vibrant and colorful) spilled on the floor.	Did Robert change?
Garden-Path	While the barber shaved the customer (that was hurried and impatient) left the shop.	Did the barber shave?
Garden-Path	As the CEO relocated the store (that was small and unsuccessful) held a sale.	Did the CEO relocate?
Garden-Path	As the gardener showered the flowers (that were yellow and blue) were gathered by a child.	Did the gardener take a shower?
Garden-Path	As the jockey settled down the horse (that was sleek and brown) stood in the stall.	Did the jockey settle down?
Garden-Path	As the model covered up the portrait (that was colorful and exact) fell from the easel.	Did the model cover herself up?
Garden-Path	While the nanny stripped the girl (that was tearful and fussy) threw a tantrum.	Did the nanny strip?
Garden-Path	While the sculptor covered up the statue (that was chiseled and perfect) stood erect.	Did the sculptor cover himself up?
Garden-Path	While the squirrels relocated the acorns (that were brown and ripe) fell from the trees.	Did the squirrels relocate?

Sentence Type	Stimulus Sentence	Comprehension Question
Garden-Path	As the trainer dried off the dog (that was playful and friendly) fetched the stick.	Did the trainer dry himself off?
Garden-Path	While Dave lied down the tiles (that were detailed and pricey) were cleaned by the maid.	Did Dave lie down?
Garden-Path	As Molly undressed the teddy bear (that was plush and huggable) lost a button.	Did Molly undress herself?
Garden-Path	While the baby-sitter woke up the infant (that was tiny and fragile) cried in his crib.	Did the baby-sitter wake up?
Garden-Path	While the butcher prepared the meat (that was tender and succulent) went through the grinder.	Did the butcher prepare himself?
Garden-Path	While the farmer settled down the pig (that was pink and squealing) escaped from its pen.	Did the farmer settle down?
Garden-Path	While the jockey groomed the horse (that was wild and testy) paced in the stall.	Did the jockey groom himself?
Garden-Path	While the manager worked out the contract (that was beneficial and generous) was signed.	Did the manager work out?
Garden-Path	While the mother undressed the baby (that was bald and helpless) cried softly.	Did the mother undress herself?
Garden-Path	As the prince disrobed the courtesan (that was graceful and voluptuous) poured the wine.	Did the prince disrobe?
Garden-Path	As the servant bathed the king (that was arrogant and pompous) ate chocolate.	Did the servant bathe?
Garden-Path	While the thief hid the jewelry (that was elegant and expensive) sparkled brightly.	Did the thief hide?
Garden-Path	As the woman soaked the shirt (that was clean and folded) sat on the dresser.	Did the woman soak herself?
Relative-Clause	The bully who the nerd (that was smart and young) challenged began the quarrel with an insult.	Did the bully start with a complement?
Relative-Clause	The committee who the applicant (that was competitive and ideal) met explained the reasoning for their decision.	Did the committee neglect to give a reason?
Relative-Clause	The expert who the source (that was renowned and skilled) revered wrote a commentary on natural selection.	Did the expert write a poem?

Sentence Type	Stimulus Sentence	Comprehension Question
Relative-Clause	The journalist who the editor (that was hardworking and vigilant) complimented revised the article for the newspaper.	Did the journalist work for a newspaper?
Relative-Clause	The diplomat who the congressman (that was untrustworthy and apathetic) insulted ended the negotiations on the spot.	Did the negotiations go well?
Relative-Clause	The plumber who the janitor (that was bored and tired) frustrated lost the key on the street.	Did the plumber lose the key?
Relative-Clause	The trumpeter who the drummer (that was brilliant and modest) loved formed the band two years ago.	Did the trumpeter start the band?
Relative-Clause	The mobster who the dealer (that was shady and dangerous) attacked organized some crimes in New York.	Was the dealer attacked?
Relative-Clause	The contestant who the host (that was famous and boisterous) offended ruined the show for the audience.	Was the show a total success?
Relative-Clause	The physician who the cardiologist (that was busy and pre-occupied) consulted checked the files in his office.	Were the files checked in the office?
Relative-Clause	The clerk who the director (that was serious and stern) disliked typed the letter to the administration.	Would the administration be receiving a letter?
Relative-Clause	The farmer who the expert (that was outgoing and enthusiastic) questioned promoted the product at the fair.	Would the product be promoted on TV?
Relative-Clause	The waiter who the cook (that was young and innovative) invited tasted the sauce for the meat.	Did someone sample the meat?
Relative-Clause	The co-worker who the professional (that was careful and punctual) intimidated delayed his response to the question.	Did the co-worker answer right away?
Relative-Clause	The carpenter who the electrician (that was careless and lazy) punched quit the job a week later.	Did the electrician and carpenter get along?
Relative-Clause	The host who the visitor (that was excited and adventurous) engaged described the route to the attractions.	Did the host describe how to get to the attractions?
Relative-Clause	The librarian who the teacher (that was old and cranky) angered misplaced the book from the depository.	Did the librarian keep track of the book?
Relative-Clause	The officer who the murderer (that was angry and impulsive) described told a lie about the past.	Did the officer say something that wasn't true?
Relative-Clause	The soldier who the enemy (that was misunderstood and feared) shot received a medal for the battle.	Did the soldier receive an honor?

**Sentence Type**	**Stimulus Sentence**	**Comprehension Question**
Relative-Clause	The model who the artist (that was weird and particular) approached signed the contract for a year.	Was a multi-year deal involved?
Relative-Clause	The analyst who the governor (that was liberal and popular) queried proposed some changes to the plan.	Was the analyst questioned?
Relative-Clause	The salesman who the cashier (that was clueless and naïve) resented mislabeled the products in the brochure.	Was there an error in the brochure?
Relative-Clause	The accountant who the statistician (that was rigorous and sharp) advised calculated the costs of the project.	Were the professionals working together?
Relative-Clause	The actor who the starlet (that was stunning and confident) respected forgot the lines during the scene.	Were they getting ready for a music concert?
Relative-Clause	The banker who the chairman (that was wealthy and greedy) informed invested a million in a start-up.	Did someone refuse to invest money?
Relative-Clause	The client who the retailer (that was pushy and loud) contacted offered a deal of the century.	Did the client retract a deal?
Relative-Clause	The detective who the spy (that was sneaky and quiet) recognized crossed the street at the light.	Did the detective cross at the sign?
Relative-Clause	The hairdresser who the beautician (that was hip and trendy) hired transformed the salon for the better.	Did the hairdresser improve the salon?
Relative-Clause	The hero who the villain (that was evil and scary destroyed) spent daylight hours in his lair.	Did the hero spend the day in the city?
Relative-Clause	The legislator who the senator (that was friendly and kind) visited falsified the documents for the trip.	Did the legislator use fake documents?
Relative-Clause	The scientist who the technician (that was diligent and meticulous) aided assumed the blame for the error.	Did the scientist blame himself for the error?
Relative-Clause	The waitress who the bartender (that was energetic and friendly) hugged dropped the tray on the floor.	Did the waitress drop the tray on the table?
Relative-Clause	The burglar who the policeman (that was decorated and revered) wounded reloaded the revolver in a hurry.	Was someone injured?
Relative-Clause	The student who the professor (that was wise and respected) trusted answered the question about the experiment.	Was there a discussion about research?
Relative-Clause	The priest who the nun (that was considerate and giving) thanked founded the shelter near the church.	Were the priest and nun involved in charity?

Sentence Type	Stimulus Sentence	Comprehension Question
Relative-Clause	The mathematician who the physicist (that was intelligent and theoretical) addressed offered the proof at the conference.	Were the scientists working in someone's office?
Relative-Clause	The investigator who the cop (that was experienced and well-known) closed the case without an arrest.	Did someone eventually get convicted?
Relative-Clause	The celebrity who the athlete (that was strong and fast) admired won the award at the ceremony.	Did the celebrity suffer a defeat?
Relative-Clause	The critic who the writer (that was controversial and acclaimed) acknowledged discussed the strengths of the piece.	Did the critic discuss the weakness?
Relative-Clause	The employee who the executive (that was rich and powerful) praised finished the project right on time.	Did the employee finish on time?
Relative-Clause	The guitarist who the band (that was loud and intense) recommended recorded the song for the album.	Did the guitarist recommend the band?
Relative-Clause	The lecturer who the dean (that was opinionated and impolite) provoked left the university in the summer.	Did the lecturer leave during the summer?
Relative-Clause	The pharmacist who the assistant (that was pretty and sincere) helped placed the order for the drug.	Did the pharmacist order a coupon book?
Relative-Clause	The reporter who the cameraman (that was funny and carefree) followed damaged the equipment during the trip.	Did the reporter break equipment?
Relative-Clause	The babysitter who the parent (that was young and single) liked planned a trip to Puerto Rico.	Was someone getting ready for a vacation?
Relative-Clause	The official who the manager (that was timid and unconfident) harassed questioned the policy of lowering wages.	Was the policy being challenged?
Relative-Clause	The violinist who the cellist (that was skilled and talented) flattered played a piece from the symphony.	Was the violinist flattered?
Relative-Clause	The medic who the doctor (that was gentle and friendly) assisted borrowed the instrument for the surgery.	Were the medical professionals preparing for an operation?
